# Effects of Physiological Loading from Patient-Derived Activities of Daily Living on the Wear of Metal-on-Polymer Total Hip Replacements

**DOI:** 10.3390/bioengineering12060663

**Published:** 2025-06-16

**Authors:** Benjamin A. Clegg, Samuel Perry, Enrico De Pieri, Anthony C. Redmond, Stephen J. Ferguson, David E. Lunn, Richard M. Hall, Michael G. Bryant, Nazanin Emami, Andrew R. Beadling

**Affiliations:** 1 Division of Machine Element, Department of Engineering Sciences and Mathematics, Luleå University of Technology, 97187 Luleå, Sweden; 2 School of Engineering, University of Birmingham, Birmingham B15 2TT, UKa.r.beadling@bham.ac.uk (A.R.B.); 3 Institute for Biomechanics, ETH Zürich, 8092 Zürich, Switzerland; 4 NIHR Leeds Biomedical Research Centre, Leeds LS7 4SA, UK; a.redmond@leeds.ac.uk (A.C.R.);; 5 Institute for Rheumatic and Musculoskeletal Medicine, University of Leeds, Leeds LS7 4SA, UK; 6 Carnegie School of Sport, Leeds Beckett University, Leeds LS6 3QS, UK

**Keywords:** total hip replacement, activities of daily living, physiological loading, joint simulator testing

## Abstract

The current pre-clinical testing standards for total hip replacements (THRs), ISO standards, use simplified loading waveforms that do not fully replicate real-world biomechanics. These standards provide a benchmark of data that may not accurately predict in vivo wear, necessitating the evaluation of physiologically relevant loading conditions. Previous studies have incorporated activities of daily living (ADLs) such as walking, jogging and stair negotiation into wear simulations. However, these studies primarily used simplified adaptations that increased axial forces and applied accelerated sinusoidal waveforms, rather than fully replicating the complex kinematics experienced by THR patients. To address this gap, this study applied patient-derived ADL profiles—jogging and stair negotiation—using a three-station hip simulator, obtained through 3D motion analysis of total hip arthroplasty patients, processed via a musculoskeletal multibody modelling approach to derive realistic hip contact forces (HCFs). The results indicate that jogging significantly increased wear rates compared to the ISO walking gait waveform, with wear increasing from 15.24 ± 0.55 to 28.68 ± 0.87 mm^3^/Mc. Additionally, wear was highly sensitive to changes in lubricant protein concentration, with an increase from 17 g/L to 30 g/L reducing wear by over 60%. Contrary to predictive models, stair descent resulted in higher volumetric wear (8.62 ± 0.43 mm^3^/0.5 Mc) compared to stair ascent (4.15 ± 0.31 mm^3^/0.5 Mc), despite both profiles having similar peak torques. These findings underscore the limitations of current ISO standards in replicating physiologically relevant wear patterns. The application of patient-specific loading profiles highlights the need to integrate ADLs into pre-clinical testing protocols, ensuring a more accurate assessment of implant performance and longevity.

## 1. Introduction

Total hip arthroplasty (THA) is the most common surgical intervention to treat hip osteoarthritis and accounts for 93.2% of implant cases [1]. While THA demonstrates high short- and medium-term success, implant survivorship declines over time, with 90% survival at 15 years, 75% at 20 years, and 58% at 25 years [2]. As younger, more active patients increasingly undergo THA [1], implants are subjected to greater mechanical stress, necessitating improved durability and performance. This heightened demand places greater importance on pre-clinical testing to ensure long-term implant reliability and prevent premature failure [3]. Younger patients, in particular, face a higher risk of implant wear and revision surgery, with optimal bearing combinations varying significantly between individuals [4,5,6,7,8].

ISO 14242-1 [1] is the primary standard for pre-clinical wear testing of THA implants. It applies stylised, sinusoidal kinematic waveforms at a fixed 1 Hz frequency, with a Paul-type double-peaked axial force profile representing both the stance and swing phases of a gait cycle [9]. While ISO provides a repeatable benchmark for implant evaluation, its simplified assumptions fail to capture the variability of real-world biomechanics, where walking speed, pause durations, and force directions vary significantly [10].

As a result, wear patterns observed in clinical retrieval studies often differ from those generated under ISO testing, suggesting that standardised test conditions may not accurately reflect in vivo implant behaviour [11,12,13,14]. Although a more extensive pre-clinical testing regime incorporating clinically relevant loading has been proposed, it has yet to be fully adopted in standardised testing protocols [15]. Similarly, computational models, such as finite element analysis (FEA), highlight discrepancies by showing patient-specific variations in contact pressures, wear zones and implant kinematics, which ISO waveforms fail to address [16,17].

The limitations of ISO testing were underscored by retrieval studies of metal-on-metal (MoM) implants, which revealed widespread issues such as tissue necrosis, metal ion release, and implant loosening outcomes linked to poor bearing surface performance and unanticipated failure mechanisms, such as edge loading [18]. Originally designed for metal-on-polymer (MoP) bearings, ISO protocols were not equipped to evaluate the unique challenges posed by MoM and ceramic-on-ceramic (CoC) implants. This underscores the risks of relying on outdated frameworks, when evaluating novel materials, as modern designs continue to transition into the market [13,19,20].

In response, ISO 14242-4 [21] was introduced to simulate adverse conditions, such as edge loading and increased inclination angles, mimicking in vivo implant misalignment and joint laxity [22]. These adverse conditions are induced by mechanisms like microseparation, where the femoral head and acetabular cup undergo lateral translation, creating small separations due to clinical variations or soft tissue imbalances [11]. Edge loading introduces additional failure modes, such as rim cracking, which has been observed in clinical retrievals of MoP bearings [23,24,25,26]. However, the introduction of crosslinked polyethylene (XLPE) has significantly reduced wear in simulator studies [27,28], with XLPE showing resistance to both edge loading [29] and increased inclination angles [30].

While adverse testing protocols offer valuable insights [29,31,32,33,34,35], they only simulate specific extreme cases and do not reflect the full range of activities experienced during daily life [36]. For example, CoC bearings develop stripe wear under minimal misalignment, with lateral mismatches as small as 0.4 mm leading to significant wear [37]. Even within adverse testing, inconsistencies arise depending on the method of edge loading applied; for instance, some studies report that edge loading via the inclination angle has no significant effect on CoC wear [30], further highlighting the need for more comprehensive evaluation methods.

With the increasing scrutiny of pre-clinical testing [38], edge loading alone should not be considered a sufficient adverse loading condition, as it does not adequately represent the physiological hip contact forces that implants experience during activities of daily living. Instead, there is a pressing need for physiologically representative testing protocols to evaluate the long-term performance of implant materials under conditions that mimic real-life scenarios.

The physiologically relevant profiles used in this study enable visualisation of biomechanical interactions and wear trends that are not predicted by ISO testing, highlighting the need to revise current standards to include activities of daily living (ADLs) profiles for a more accurate assessment of implant behaviour under real-world conditions.

Despite a growing body of literature on computational and adverse condition testing, no prior experimental study has applied realistic, patient-derived loading profiles—captured from actual THR patients during ADLs—within a standardised hip simulator framework. This study introduces the novel integration of musculoskeletal modelling with hip simulator-based wear assessment to demonstrate that current ISO standards may substantially underestimate wear under real-world conditions. It also highlights the influence of lubricant protein concentration, offering physiological realism, often overlooked in standardised protocols. These findings collectively underscore the urgent need to revise pre-clinical testing frameworks to incorporate real-world kinematics and loading conditions, thereby improving the reliability of THR performance evaluations.

The aim of this study is to evaluate the wear behaviour of metal-on-polymer (MoP) total hip replacements under physiologically relevant loading conditions derived from patient-specific activities of daily living (ADLs), including jogging and stair negotiation. By applying experimentally measured hip contact forces through a three-station hip simulator, this study seeks to identify how deviations from standard ISO profiles influence wear rates, frictional torque, and overall bearing performance. The findings intend to inform the development of more representative pre-clinical testing protocols for MoP bearings, which remain the most ubiquitous configuration in clinical practice [1,39].

## 2. Materials and Methods

Clinical 32 mm diameter Cobalt Chrome Molybdenum (CoCrMo) heads (Aesculap, Tuttlingen, Germany) were paired with moderately crosslinked (50 kGy) ultra-high-molecular-weight polyethylene UHWMPE (XLPE) acetabular liners (Aesculap, Germany) for wear and frictional torque testing. The acetabular liners were soaked in DI water for three weeks prior to testing. The mass loss was determined gravimetrically every week according to ISO 14242-2 [40].

### 2.1. Wear Test

**Figure 1:**
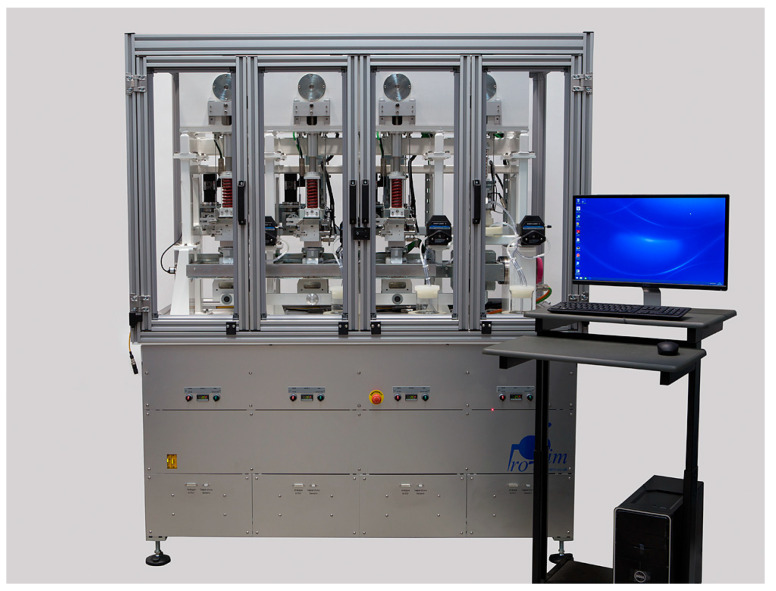
A three-station Prosim Hip wear simulator (Simulation Solutions, UK) combined with a load soak.

Three metal-on-XLPE bearings were evaluated in a three-station Prosim Hip wear simulator (Simulation Solutions, Stockport, UK) combined with a load soak shown in Figure 1. The CoCrMo femoral heads were mounted onto femoral spigots tapered to the part design, with the acetabular liners secured to the acetabular fixation with a matching titanium shell (Aesculap), cemented into PEEK fixtures to ensure a correct centre of rotation for the bearing. The fixtures were designed to enable removal of components for gravimetric wear assessment. The cup fixtures were set at an inclination angle of 30 degrees, to match the requirements of ISO 14242-1 [9].

An initial test of 5 million cycles of the ISO 14242-1 gait profile was previously completed on the same full-ISO hip simulator (SimSol, Stockport, UK), conducted using foetal bovine serum (FBS) diluted to 25% (*v*/*v*) (17 g/L) concentration with deionised water and sodium azide (0.03% *w*/*v*) complying with ISO 14242-1 [9] to compare with historical data.

A second test was conducted, on the same full-ISO hip simulator (SimSol, UK), for this report, using bovine calf serum diluted to 30 g/L protein concentration, supplemented with 0.03% (*w*/*v*) sodium azide to retard bacterial growth. The testing sequence, summarised in Table 1, comprised an initial 2 million cycles of ISO standard walking to allow the bearings to run-in, followed by the application of ADLs applied consecutively to the same run-in components.

In the first test, foetal bovine serum (FBS) was diluted to 17 g/L (25% *v*/*v*) protein concentration, consistent with historical ISO 14242-1 standards. In the second test, BCS was used at a 30 g/L protein concentration, aligning with the current ISO requirement of 30 ± 2 g/L [9]. This adjustment reflects literature findings that protein concentrations below 20 g/L do not replicate clinically relevant wear patterns [41].

For both tests, the components were weighed, and serum was changed every 0.5 Mc according to ISO 14242-2. The parts were gravimetrically measured, according to ASTM F2025-06 [42], using a Mettler Toledo XP205 (Mettler-Toledo GmbH, Greifensee, Switzerland) analytical balance. The polymeric parts were then put on soak overnight to ensure parts were not dry when initialising the next phase. The load soak control was used as a correction for fluid uptake of the components. Due to the reduced frequency of the stair profiles, shown in Table 2, determined from patient-derived data [36], the system lubricant was refreshed every 0.25 Mc for the last 1 Mc of testing. Statistical significance was tested using a one-way analysis of variance (ANOVA).

### 2.2. Profiles

**Figure 2:**
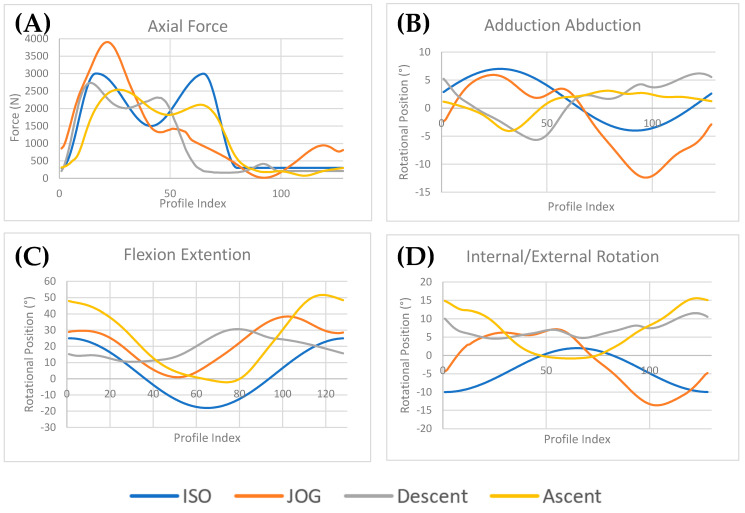
Comparison of hip simulator input profiles for (**A**) axial force, (**B**) adduction–abduction, (**C**) flexion–extension, and (**D**) internal/external rotation–flexion.

To approximate the forces experienced by a joint replacement, Lunn et al. [36] analysed a cohort of 132 THR patients, collecting motion capture and force plate data across various activities in a gait analysis lab; of those patients, 49 completed stair ascent and 47 completed stair descent, with an average weight of 71.5 Kg, with 4 patients performing jogging, with an average weight of 78.1 kg. The data was then processed through a musculoskeletal modelling analysis [36] to calculate hip joint contact forces (HCFs) and hip joint kinematics from patient motion and external ground reaction forces, shown as hip simulator input profiles in Figure 2. The data was then qualitatively compared to the Orthoload database for validation [43]. The peak values for these profiles are presented in Table 3.

The jog and dwell, stair descent, and stair ascent profiles incorporated transition phases, shown in Figure 2, during a low-loaded swing to facilitate testing. Due to a greater disparity between its start and end points, stair descent required an additional transition phase at the end of the profile. Consequently, the total cycle time for stair descent was increased from 1.44 to 1.8 s, allowing the physiological profile to run for the first 1.44 s at a physiologically relevant frequency of 0.694 Hz, followed by a 0.36 s transition phase, shown in Table 2.

Jog and jog and dwell were run as direct comparisons to see the effects of a stop-dwell-start motion, which represents a more severe scenario compared to jog, with a potential increase in static friction thought to increase the overall wear. The jog and dwell profile was run for 10 consecutive jog cycles, followed by 5 s of dwell, characteristic of the most common patient stop duration during ADLs [44].

Stair ascent was also adapted, with a −10° offset for the flexion extension wave, in accordance with the capabilities of the hip simulator (maximum of +61°) and to ensure that physical impingement of the cup and stem fixtures did not occur. This adaptation ensured that the sliding distance during the loading phase and the area of contact, due to the centre of rotation, were not affected.

### 2.3. Frictional Torque Acquisition

The forces and torques at the bearing interface were assessed using 6-axis load cells that were secured vertically above the cup. A custom MATLAB R2023b script (MathsWorks, Natick, MA, USA) developed by Pryce et al. [45], based on the methodology described by Haider et al. [46], was used to determine the frictional torques at a steady state during each test phase.

## 3. Results

### 3.1. Frictional Torque Profiles

**Figure 3:**
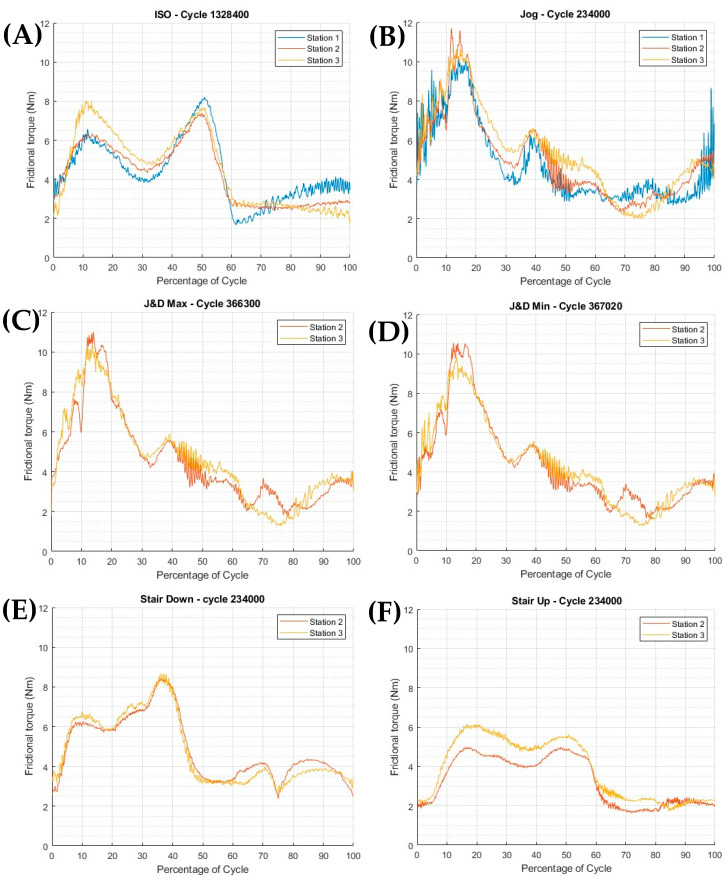
Friction torque data taken from stable points within each profile: (**A**) ISO14242; (**B**) jog; (**C**) jog and dwell, 1st jog in series; (**D**) jog and dwell, 10th jog in series; (**E**) stair descent; (**F**) stair ascent.

The peak and average peak torques from Figure 3 can be seen in Table 4, showing an increase in torque from approximately 8 Nm during ISO to just below 11 Nm for the jogging profiles. The ISO torque curve remains generally higher during the stance phase, dropping to 5 Nm before rising to 8 Nm at toe-off. Conversely, jogging profiles exhibit a substantially lower toe-off peak (~6 Nm) but exhibit higher peak torques (~11 Nm) at heel strike, highlighting a distinct shift in torque distribution compared to ISO gait patterns. The maximum torque values for the jogging profiles consistently occur during the heel strike phase, following a similar pattern across all profiles. This distinction is particularly important when comparing the jogging profile to the stop-dwell-start motion of the jog and dwell (J&D) profile.

When isolating the torque curve from the initial (1st) cycle and the final (10th) cycle of the jogging phase (J&D Max and Min, respectively, in Table 4), the torque shows a minimal reduction from 10.71 Nm to 10.20 Nm, indicating a negligible effect of the dwell period on torque during the jogging phase. During the jogging profile, station 1 was lost in the final stages due to a leakage issue. Means and standard deviations were calculated based on samples n = 3 for phases 0–3 Mc and n = 2 for 3–5 Mc following the exclusion of one station due to fluid leakage.

For the stair profiles, the descent peak torque occurs at the beginning of the stance phase, with the ascent peak torque occurring at the end of the stance phase. The values of peak torque share a positive correlation with the wear volume produced within each profile. The impact of the heel strike phase within each profile can be viewed as a percentage of time for the profile to reach heel strike, as shown in Table 5. The stair descent profile is the only profile where the maximum torque does not occur when the maximum axial force is applied.

### 3.2. Wear of ISO vs. Jogging

**Figure 4:**
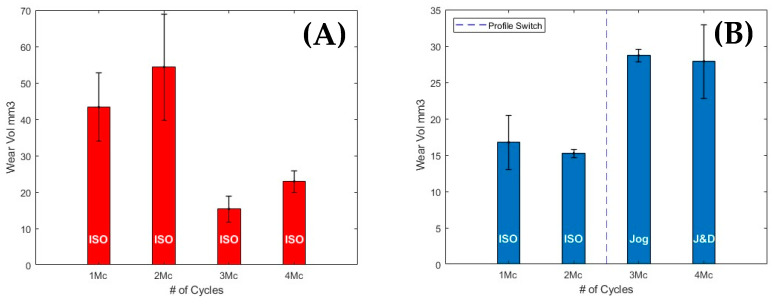
Wear comparison trends of (**A**) a continuously run ISO 14242 gait, using 17 g/L FBS for 4 Mc, and (**B**) 2 Mc activities of daily living, jog, and jog and dwell following 2 Mc of ISO 14242 gait, totalling 4 Mc using 30 g/L BCS.

A standardised ISO test was conducted, as shown in Figure 4A, showing the wear volume trend over 4 Mc using 17 g/L protein concentration, with a running-in phase of 2 Mc, followed by a drop in wear volume of around 50% where steady-state wear is formed. This highlights the biphasic wear pattern of a standard ISO wear test on a MoP bearing.

The significant decrease (*p* < 0.05) in wear volume between Figure 4A,B is attributable to the change in lubricant protein concentration, with wear reducing from 43.3 ± 9.4 mm^3^ to 16.7 ± 3.7 mm^3^ at 1 Mc, and from 54.3 ± 14.6 mm^3^ to 15.2 ± 0.6 mm^3^ at 2 Mc, when moving from 17 g/L FBS to 30 g/L BCS.

Figure 4B denotes the study of activities of daily living, with the first 2 Mc run under ISO walking gait conditions to ensure the running-in period was met. From 2 Mc to 3 Mc, the physiologically relevant profile of patient jogging was applied, significantly increasing the wear by almost two-fold from 15.99 ${mm}^{3}$ to 28.28 ${mm}^{3}$ per Mc (*p* < 0.05). From 3 Mc to 4 Mc, a jogging and dwelling profile was applied to the bearings, outputting similar wear—slightly decreased but higher in standard deviation—to that of a continuous jogging profile. A breakdown of the wear rates can be seen in Table 4.

Based on the wear trend observed in Figure 4A for the standard ISO test, Figure 4B reveals a reversal in the expected wear pattern, when the ADLs of jogging are applied, producing a significant increase in wear at 3 Mc. It is important to note that the testing conditions, equipment, and materials remained consistent across both tests, with the only variable being a change in lubricant.

### 3.3. Stair Negotiation

**Figure 5:**
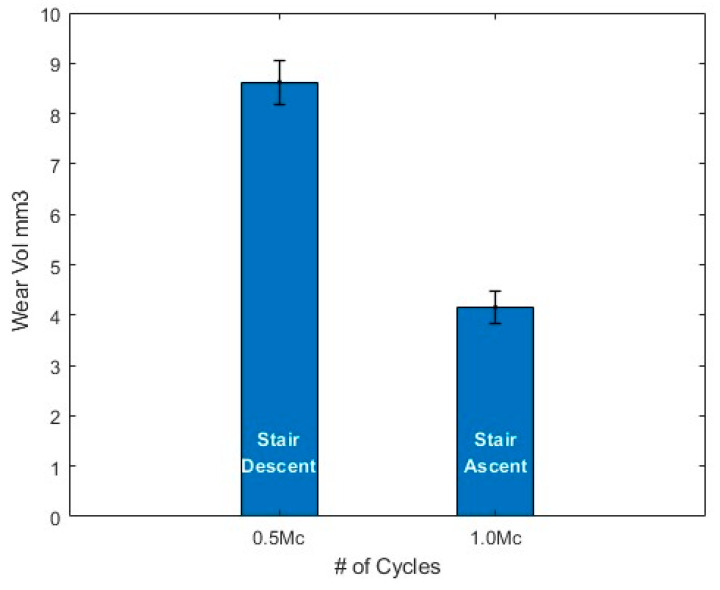
Volumetric wear rate of activities of daily living of 0.5 Mc descending and 0.5 Mc ascending stairs, completed over the last 1 Mc of the 5 Mc test. Stair descent from 4.0 to 4.5 Mc and ascent from 4.5 to 5.0 Mc using BCS at a 30 g/L concentration.

The ADLs of stair descent and ascent were applied to the bearings for the last million cycles of the test, split into 0.5 Mc for each profile. The wear volumes accrued are presented in Figure 5, revealing a two-fold increase in wear volume for stair descent (8.6 ± 0.43 ${mm}^{3}$) compared to stair ascent (4.2 ± 0.31 ${mm}^{3}$), a substantial but not statistically significant difference (*p* = 0.071), likely attributable to the reduced sample size (n = 2) in the final phase.

## 4. Discussion

The current study uses hip contact forces (HCFs) from Lunn et al. [36], who assessed 47 patients for stair descent, 49 for stair ascent, and 4 patients for jogging. Previous datasets [17,47,48,49,50,51] and physiological parameters have analysed HCFs enacted from activities of daily living, derived from THRs. These studies all conclude that a better understanding of the in vivo kinematics is required to better replicate and predict the wear of THA patients.

Adverse testing of ADLs has been limited by the simulator capability and the challenges of applying precise HCFs to accurately replicate in vivo conditions [52]. As a result, computational studies have modelled the effects of ADLs, with simulations predicting an increase in wear [16,17] for these profiles compared to standard ISO gait test profiles, which fail to account for localised changes in acceleration [53]. Unlike continuous ISO test profiles, ADLs are inherently sequential and include periods of no motion, a phenomenon commonly overlooked in hip simulator testing [54]. Studies have shown that bearing friction increases when motion restarts after a dwell period, with HoH bearings such as MoM [55,56] exhibiting significantly higher increases compared to MoP bearings [44,57].

During the ISO gait phase of the test, peak torque was maintained at around 8 Nm (see Figure 3A) and stayed at this high peak over a larger portion of the cycle when compared to the jog (see Figure 3B). Once the profile was switched to jog, a change in pattern was seen with the peak torque increasing to 11–12 Nm; this was repeated when the profile was switched to J&D (see Figure 3C,D). This torque curve pattern and peak torque led to a similar wear rate across the two million cycles of the jogging profiles.

The representative wear rates are shown in Figure 4B, in comparison to the continuous ISO test (Figure 4A). A switch to the jogging gait profiles led to a significant increase in frictional torque (Figure 3A,B), which directly correlated with an increase in wear volume. However, since frictional torque remained stable across both jogging profiles, the wear rate was also maintained. This finding aligns with a previous start-dwell-stop study by Hadley et al. [58], using MoP bearings, where only a slight increase in wear rate was observed for a 10-walking, 5-dwell divided profile.

This behaviour can be attributed to MoP bearings operating primarily in boundary lubrication [29,59], demonstrating greater resilience in HoS bearings. Unlike HoH bearings, MoP bearings are less dependent on fluid load support [35] and, therefore, do not experience a significant increase in wear when the lubrication regime is depleted.

Daily activity testing was also conducted by Affatato et al. [60], but no comparison to ISO wear rates was given. Previous studies have also physically assessed the effects of jogging on a hip simulator, such as the study by Bowsher et al. [61]. Using an orbital simulator, they found an increase in wear for jogging at 1.75 Hz, contrasting with Viitala et al. [62], who found an decrease in wear for jogging at 1.25 Hz, but with significantly larger bearings (54 vs. 28 mm). Both studies [61,62] applied the current practice of simulation by mapping the axial force applied to the hip joint and an acceleration of the sinusoidal ISO profiles to physiologically relevant frequencies. This application of profiles is not physiologically accurate and does not take into consideration the kinematics within a clinically relevant gait profile, thereby leading to a discrepancy in the torques and reported wear data.

Switching to physiologically relevant gait profiles not only alters peak axial forces but also significantly shifts torque distribution throughout the cycle. Jogging was expected to impose a harsher loading profile; however, instead of a uniformly increased torque curve, it produced a single larger peak at heel strike while maintaining a much lower torque throughout the swing phase, compared to an ISO gait. This suggests that wear is not solely dictated by peak axial forces but is instead influenced by the dynamic nature of kinematics across the gait cycle.

### 4.1. FBS vs. BCS

The decrease in wear volume with increasing protein concentration observed in Figure 4 is consistent with the previous literature [63,64], which reports that higher protein content reduces friction in MoP bearings [65]. Ideally, the lubricant’s protein content in hip simulator studies should match that of THA patients’ synovial fluid. However, achieving this remains a challenge due to the wide variability in reported protein concentrations, ranging from 20–35 g/L in healthy patients [66] to ~30 g/L in osteoarthritis patients [67]. Nonetheless, simply matching total protein content does not ensure realistic lubrication conditions, as the composition of individual protein constituents also affects wear. This was demonstrated by Alpha/Beta calf serum (ACS/BCS) lubricants, both assessed at 20 g/L, yielding different wear rates [68]. More reproducible wear data has been achieved using dilution methods that account for protein content and albumin/globulin (A/G) ratios (Wang et al., 2004 [69]).

Kaddick & Wimmer demonstrated that the wear rates of PE bearings do not significantly differ when using FBS or BCS at the same protein concentration [70], with both lubricants showing biphasic wear trends. Their findings highlight the variability in wear that can occur due to changes in protein concentration, reinforcing the importance of maintaining consistency in lubricant composition to ensure reliable wear data. This study further demonstrates that an increase in protein concentration from 17 g/L (FBS) to 30 g/L (BCS) leads to a significant decrease in wear volume, emphasising the influence of protein concentration on wear outcomes.

Due to a lack of consensus on the optimal protein concentration for synovial fluid [71], maintaining consistency in lubricant protein content is critical in current and future hip simulator studies. While the literature suggests that protein concentrations below 20 g/L do not produce clinically relevant wear mechanisms [41], previous ISO 14242 standards required a serum dilution of 25% to a minimum of 17 g/L and presently call for a serum dilution to a protein concentration of 30 ± 2 g/L, hence the difference in historic data across the literature. This study directly compares and highlights that a protein concentration of 30 g/L vs. 17 g/L (25% *v*/*v*) creates a significant decrease in the wear volume, using the same components and equipment over a long-term test. This emphasises both the importance of maintaining a consistent protein concentration and the degree of variance that can occur without it.

### 4.2. Stairs

Several computational simulations have attempted to model the wear output of physiological gait profiles using Bergmann’s [72] HCFs [73,74], Lunn’s [36,75] HCFs [16] and HCFs [76] from healthy young individuals without implants [77]. These models consistently suggest that stair ascent incurs greater wear volume than stair descent, attributing this to increased contact pressure [73,78], higher average load across the gait cycle [74], and distance travelled by the nominal contact point [16]. However, these simulations have not been physically validated using a hip simulator, and Figure 5 reveals a significantly higher frictional torque and wear volume output for stair descent compared to ascent. This discrepancy highlights that current models may undervalue the role of rapid load acceleration on implant bearings while overemphasising cumulative metrics such as mean load, range of motion, and sliding distance.

The noted simulation studies [16,73,74] have typically prioritised cumulative sliding distance and mean contact pressure, guided by Archard’s wear law. However, our findings suggest these assumptions are insufficient: the rapid load application during descent likely induces surface deformation, enlarges the contact area, and accelerates wear. This points to a potentially more accurate predictive approach underpinned by dissipated energy principles rather than static force–pressure models [79].

Recent computational studies have advocated for wear prediction frameworks that employ dissipated energy, rather than traditional pressure- and distance-based models. Toh et al. [80,81] applied this method to THR bearing surfaces and demonstrated its efficacy in simulating activity-specific wear responses. Although their studies primarily focused on walking and cycling using hip joint rotations and loadings, they did not compare ADL or ISO profiles. Nonetheless, their findings reinforce the role of dynamic factors such as acceleration, torque, and energy transfer, more accurately capturing wear mechanisms than static models. Complementary work by Feyzi et al. [82,83] extended the application of dissipated energy in combination with Archard’s principles to head–neck taper junctions, demonstrating the broader relevance of this approach across tribological implant interfaces.

Within this conceptual framework, stair descent may generate greater wear than ascent due to its combination of higher angular velocity and elevated torque demands, particularly during rapid deceleration phases. These conditions likely lead to increased energy dissipation per cycle and, by extension, greater predicted wear volume.

Additionally, the application of physiologically realistic waveforms may cause migration of the contact patch from previously bedded-in surfaces to regions of the implant that are initially unworn and less conforming. This shift may exacerbate wear in ways not predicted by conventional wear path models.

A comparison of frictional torque data during stair ascent and descent reveals distinct biomechanical patterns. Stair descent begins with initial foot contact at peak axial force, followed by limited joint rotation during the controlled lowering of the body. As the centre of mass shifts, a levered motion occurs during the pre-swing phase, culminating in toe-off, which corresponds with peak torque, before the opposite limb accepts the load. In contrast, stair ascent relies on a progressive extension of the hip, generating a more uniform torque distribution throughout the loading response and push-off phases. Consequently, impact acceleration—quantified as the time to peak axial force (see Table 5)—and maximum torque magnitudes are more pronounced in stair descent, indicative of higher transient loading. Conversely, stair ascent involves a more sustained loading profile, with greater sliding distance but lower peak torque, highlighting fundamental gait differences in mechanical demand on the prosthetic joint.

The rate of change of force for the profile is a function of the profile frequency and time to reach heel strike from the swing phase. This, alongside the applied axial force, is deemed to be the dominating factor for increasing wear. It is suggested that the increased impact force induces greater plastic deformation of the polymer bearing, thereby increasing the surface area in contact during sliding, as such increasing the wear. Whilst this is intuitive when comparing a jogging and walking gait, this effect can be seen directly by comparing the stair activities. A similar axial force is applied with both profiles, but the maximum force of the stair descent is applied at 0.14 s vs. 0.27 s for the stair ascent profile. Even with a much greater range of motion in the flexion–extension direction under the applied load, stair ascent produces a lower wear rate, due to a reduced impact of the peak torque.

### 4.3. Limitations

The frictional torque calculated within this study, using methods outlined in [46], is around four times higher than that calculated by Haider et al. [46]. A reduction in torque is also seen in Saikko et al. [84,85]. But comparable results are seen in Nečas et al. [86] and Sonntag et al. [87]. This difference is most likely due to the offset of the sensor positioning within the calculations. However, it should also be noted that there is a greater variance not only in the bearing material combinations, sizes of components, exact locations of torque sampling within the profile cycle and lubrication protein concentrations but also in the equipment used. Due to the differences in calculations, assumptions, and individual hip simulators, it would seem pertinent to only compare the frictional data within this set.

The comparison of FBS at 17 g/L and BCS serum at 30 g/L for the initial 2 Mc of the ISO walking gait profile is not a direct comparison; however, the aim of the comparison is to highlight the nature of the unreliability of the benchmark data provided by ISO testing, and that it is not suitable for comparison or representative of in situ wear.

The authors understand that these activities of daily living are still not entirely representative of real-life implant patient kinematics, but that this is a first step to moving towards a testing mandate, where the industry attempts to apply increasingly representative environments to the implants. This study shows that in situ analysis from load cell sensors can be applied, potentially to move forward to using daily living cycles, in a dynamic manner, rather than merely a continuous running of a single profile.

This study used a sequential testing approach, exposing the same set of bearings to multiple activity-specific profiles in series. While prior phases—such as jogging—may influence subsequent wear behaviour, this method has precedent in the literature. Saikko et al. [62] and Bowsher et al. [61] adopted similar multi-activity protocols, whereas Hadley et al. [58] used new components for each profile without any running-in, which may itself affect early wear. Here, an initial 2 million ISO standard cycles were applied to ensure steady-state conditions (Figure 4A), before introducing the ADL profiles. Due to time, cost, and material demands of separate tests for each activity, a sequential protocol was adopted.

While this study provides valuable insights into the effect of patient-representative daily living kinematics on wear rates, a limitation is the disparity in sample size across different activity profiles. The jogging profile was derived from only four patients, with an average body weight of 78.1 kg, whereas stair ascent and descent data were based on 49 and 47 patients, respectively, with an average weight of 71.5 kg. These differences in sample size and participant weight may influence the generalisability of the findings, particularly for jogging, where a larger and more representative dataset could further validate the observed trends. Additionally, during testing, station 1 was lost after 3 million cycles due to a leakage issue. This reduction in sample size may have introduced minor variability in long-term wear rate consistency and limited the robustness of statistical comparisons during later phases.

Considering changing regulatory requirements and following on from clinical disasters (MoM failures), it would be valuable if the industry and medical implant field were aware of the current testing procedures and protocols, to ensure that they are more representative of patient environments. The data provided in this study shows that the introduction of ‘patient-representative daily living kinematics,’ extracted from a biomechanical study of real-life patients, when applied to implant bearings, enacts a physical change in the wear rates of MoP implants.

At this juncture, the authors can only emphasise that these results have implications on how the medical implant industry conducts pre-clinical testing on these devices before implantation into patients.

## 5. Conclusions

This study demonstrates that activities of daily living, such as jogging and stair navigation, result in increased wear on MoP bearings compared to standard ISO gait profiles. Specifically, stair descent induces higher wear than ascent due to the combined effects of kinematics and the acceleration of impact forces. Furthermore, the inclusion of a dwell phase in jogging and dwelling profiles did not notably alter wear behaviour, suggesting the robustness of MoP bearings under such conditions. A higher protein concentration in lubricants (30 g/L vs. 17 g/L) significantly reduces wear rates, underscoring the importance of physiologically relevant test conditions in pre-clinical evaluations.

These findings highlight critical differences between physiologically representative and standardised ISO profiles, emphasising the need for updated testing protocols that more accurately replicate in vivo conditions. The outcomes have significant implications for improving pre-clinical testing to better predict the long-term performance and reliability of hip replacement implants.

## Figures and Tables

**Table 1:** Categorisation of activities of daily living testing.

Phase 1		Phase 2		Phase 3	
1 Mc	2 Mc	3 Mc	4 Mc	5 Mc	
ISO 14242-1	ISO 14242-1	Jog	Jog and Dwell	Stair Descent	Stair Ascent

**Table 2:** Frequency and cycle times of applied profiles.

Profile	Frequency (Hz)	Cycle Time (s)
**ISO**	1	1
**Jog**	1.4	0.727
**Jog and Dwell**	1.4	0.727
**Stair Descent**	0.694–0.556	1.44–1.80
**Stair Ascent**	0.629	1.59

**Table 3:** Maximum and minimum values of ADLs profiles including applied load and kinematic axis for the hip simulator.

	Axial Load (N)		FE °		AA °		IE °	
	Max	Min	Extension	Flexion	Abduction	Adduction	Internal Rotation	External Rotation
**ISO**	3000	300	25	−18	7	−4	2	−10
**Jogging**	3901	18	38.42	0.97	5.92	−12.39	7.16	−13.61
**Stair Descent**	2735	164	30.63	10.45	6.18	−5.67	11.52	4.58
**Stair Ascent**	2538	77	51.61	−2.14	3.10	−4.07	15.58	−0.82

**Table 4:** Wear rates per million cycles and mean peak torque values for each individual applied profile within this study, correlating to the data points in Figure 3.

	Phase 1		Phase 2			Phase 3	
	1 Mc	2 Mc	3 Mc	4 Mc		5 Mc	
**Profile**	ISO 14242-1	ISO 14242-1	Jog	Jog and Dwell		Stair Descent	Stair Ascent
**Wear rate** $\left({mm}^{3}\right)$	16.7 (±3.71)	15.2 (±0.55)	28.7 (±0.87)	27.9 (±5.05)		8.6 (±0.43)	4.2 (±0.31)
				**J&D Max**	**J&D Min**		
**Avg Max Torque (Nm)**	7.85 ± 0.35		10.88 ± 0.62	10.71 ± 0.29	10.20 ± 0.34	8.55 ± 0.12	5.56 ± 0.59

**Table 5:** Percentage of cycle and time to reach heel strike, correlating with maximum applied axial force of the profile.

Profile	ISO	Jog	Stair Descent	Stair Ascent
**% of cycle to heel strike**	10%	14%	10%	17%
**Time to heel strike (s)**	0.1	0.1	0.14	0.27

## Data Availability

The original contributions presented in this study are included in the article. Further inquiries can be directed to the corresponding authors.

## Abbreviations

HoH	Hard-on-Hard
HoS	Hard-on-Soft
MoP	Metal-on-Polymer
CoP	Ceramic-on-Polymer
MoM	Metal-on-Metal
CoC	Ceramic-on-Ceramic
THA	Total Hip Arthroplasty
THR	Total Hip Replacement
FBS	Foetal Bovine Serum
BCS	Bovine Calf Serum
ACS	Alpha Calf Serum
HCF	Hip Contact Force
ISO	ISO 14242 Gait Profile
